# Effectiveness of the Use of Polymers in High-Performance Concrete Containing Silica Fume

**DOI:** 10.3390/polym15183730

**Published:** 2023-09-11

**Authors:** Alya Harichane, Nadhir Toubal Seghir, Paweł Niewiadomski, Łukasz Sadowski, Michał Cisiński

**Affiliations:** 1 Institute of Science, University Center of Tipaza, Tipaza 42000, Algeria; toubalseghir.nadhir@cu-tipaza.dz; 2 Department of Materials Engineering and Construction Processes, Wroclaw University of Science and Technology, Wybrzeże Wyspiańskiego 27, 50-370 Wroclaw, Poland; pawel.niewiadomski@pwr.edu.pl (P.N.); lukasz.sadowski@pwr.edu.pl (Ł.S.); 3 Department of Advanced Material Technologies, Wroclaw University of Science and Technology, Wybrzeże Wyspiańskiego 27, 50-370 Wroclaw, Poland; 240524@student.pwr.edu.pl

**Keywords:** polycarboxylate ether superplasticizer (PCE), rheology, molecular architecture, high performance concrete, carboxylic groups

## Abstract

The incorporation of polycarboxylate ether superplasticizer (PCE)-type polymers and silica fume (SF) in high-performance concretes (HPC) leads to remarkable rheological and mechanical improvements. In the fresh state, PCEs are adsorbed on cement particles and dispersants, promoting the workability of the concrete. Silica fume enables very well-compacted concrete to be obtained, which is characterized by high mechanical parameters in its hardened state. Some PCEs are incompatible with silica fume, which can result in slump loss and poor rheological behavior. The main objective of this research is to study the influence of three types of PCEs, which all have different molecular architectures, on the rheological and mechanical behavior of high-performance concretes containing 10% SF as a partial replacement of cement. The results show that the carboxylic density of PCE has an influence on its compatibility with SF.

## 1. Introduction

According to the World Cement Association (WCA), the cement industry is responsible for 5–6% of global carbon dioxide emissions [1]. This association requires a significant reduction in the carbon footprint of the cement industry. One approach to this is to partially move away from cement in favor of “mineral additions”, which can often be waste products from other industries. However, substituting cement with mineral additions such as silica fume strongly modifies the rheological and mechanical properties of concrete [2,3]. Therefore, in some cases, the composition of concrete mixes has to be adjusted with regard to these additives in order to maintain the quality and parameters of the concrete. The use of polycarboxylate ether superplasticizers (PCEs) is absolutely necessary for concretes that contain mineral additives, e.g., silica fume, in order to make these additives “work”. The compatibility of PCEs with silica fumes is currently the subject of many studies [4,5,6,7]. 

PCEs are currently the most effective at reducing the water content (up to 40%) and improving the workability and fluidity of concrete compared to other types of superplasticizers. PCEs are copolymers and have a comb structure [8,9]. The main chain of a PCE carries a negatively charged carboxylic group (COO–), which promotes the adsorption of the polymer onto the positively charged surfaces of cement particles or individual hydrate phases, primarily ettringite [Ca_3_Al(OH)_6_ 12H_2_O]_2_(SO4)_3_ 2H_2_O [10]. This occurs based on the electrostatic interaction. While adsorbed, the polymer promotes the dispersion of the cement particles due to the steric hindrance produced by its side chains containing polyethylene glycol (PEG) structures [11,12,13,14,15,16,17,18,19,20,21]. As a result, the water entrapped in the agglomerated binder particles can be released, increasing the flowability and reducing the viscosity of cement paste [22,23,24,25].

The adsorption and dispersing capacity of PCEs in cement-based materials depends on their chemical structure as well as their molecular weight, carboxylic groups, density, and length of the side chain [26,27,28]. Some PCEs are incompatible with silica fume in high-performance concretes, which in turn can result in a loss of slump and poor rheological behavior. This is due to the prior adsorption of the PCE on the surface of the silica fume [22,29,30,31], increasing the dispersion of the SF particles. Therefore, the adverse effect of PCEs on cement hydration could be mitigated by adding SF, which provides additional surfaces for PCE absorption and, as a result, reduces PCE adsorption on cement surfaces [6].

To the best of our knowledge, the effectiveness of PCEs in high-performance concrete (HPC) containing silica fume (SF) as a partial replacement of cement has not been fully investigated. There are several knowledge gaps regarding this matter, especially concerning the interaction of SF and PCE (with different molecular weights and carboxyl densities) in HPC. To fill this research gap, the authors of this paper conducted studies of the interaction between PCE and silica fume in cement slurries as well as in high-performance concretes (HPC) that have a low w/b ratio. Three PCEs with different molecular compositions were used to study their impact on the properties of concrete (HPC). Therefore, the fluidity and mechanical properties of HPC were evaluated. The impact of the PCE on the shear stresses, plastic viscosity, and zeta potential of cement slurries (containing 10% silica fume as a cement substitute) was also measured.

## 2. Materials and Methods

### 2.1. Materials

#### 2.1.1. Superplasticizer

Three types of superplasticizers produced in Algeria were used: Polyflow SR 3600—designated as PCE1, Polyflow SR 5400—designated as PCE2, and Medaflow 30—designated as PCE3. The three polymers are new-generation, non-chlorinated, and based on modified polycarboxylic ether. The molecular weight (Mn, Mw) and the polydispersity indices (PDI) of the superplasticizers were measured using multi-detector steric exclusion chromatography (SEC) in France (laboratory for catalysis, polymerisation; processes and materials C2P2). The analytical conditions are presented in Table 1. The characteristics of the superplasticizers, according to their technical data sheets, are presented in Table 2.

#### 2.1.2. Portland Cement and Silica Fume

**Figure 1:**
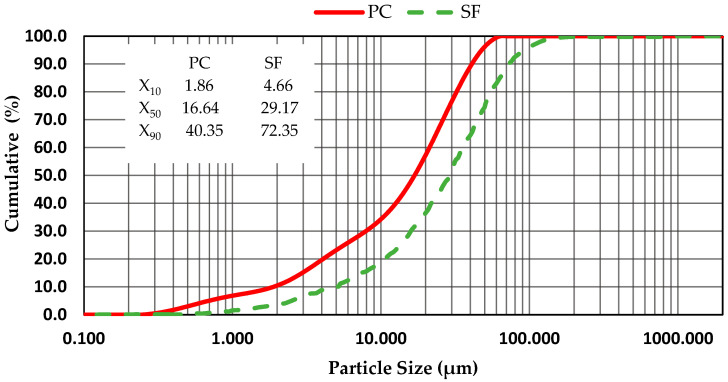
Particle size distribution curves of the PC and SF.

The Portland cement (PC) that was used in the experimental study was CEM I 52.5 R. It is commercially produced by the Lafarge Company, whose manufacturing site is located in the city of Msila (in the eastern region of Algeria). The microsilica (SF) sample was obtained from Granitex, Algeria. Its marketing name is Medaplast HP, and its specific surface is 23,000 m^2^/kg. The chemical composition of the PC and SF is presented in Table 3. The particle size distributions of the PC and SF were prepared using a Malvern Mastersizer 2000 analyzer (liquid:Hydro, 2000MU). The results are presented in Figure 1.

#### 2.1.3. Aggregates

**Figure 2:**
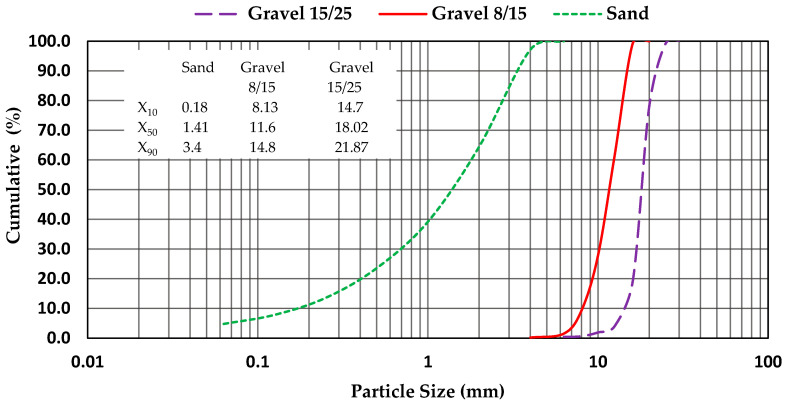
Particle size distribution curves of the used aggregates.

The properties of the aggregates are summarized in Table 4, and their particle size distribution curves are given in Figure 2.

### 2.2. Experimental Measurements

#### 2.2.1. Evaluation of the Content of Carboxyl Groups Using the Titration Method

The acid-base titration method was used to measure the content of carboxyl groups in each polymer sample [32]. The measurements involved the determination of the amount of NaOH solution, which in turn was expressed as the product of its molar concentration and the volume that was needed to titrate a sample. The determined amount of NaOH (number of moles) is directly related to the content of carboxyl groups in polymers because COO– and Na^+^ ions, which come from carboxyl groups and NaOH, react with each other in a stoichiometric ratio. Therefore, the more NaOH needed to titrate the sample, the more carboxyl groups it contains. The content of carboxyl groups can then be calculated as follows:

C(COO–) = [CNaOH × VNaOH] × m/100

where

C(COO–) = content of carboxyl groups (mmol/100 g);
CNaOH = molar concentration of NaOH (mmol/L);
VNaOH = volume of the NaOH solution used (L); and m = weight of the sample.

#### 2.2.2. The Composition of the Concrete Mix and the Performances of the Polymers in the High-Performance Concrete (HPC)

The recipe of the concrete was determined on the basis of the Dreux–Gorisse method while taking into account the ratio of its components (water, cement, sand, and aggregates), as shown in Table 5. The prepared concrete had a mechanical compressive strength of 55 MPa and a slump of 175 mm. The manufactured concrete mixes had a constant water-to-binder ratio of 0.35. The amount of PCE added to the concrete (in the presence of SF) in relation to the binder was adopted based on viscosimetric measurements (using a VT 550 viscometer). The ratio was assumed to be PCE/b = 1.75%.

#### 2.2.3. Fluidity

The slump test was used to evaluate the fluidity of the concrete mix in the case of all the formed HPCs. The dosage of superplasticizer (PC/b) was 1.75%, and it was determined using the VT 550 viscometer. The initial fluidity of the HPC was measured 5 min after mixing all the components. The concrete mix was then placed in a container, which was covered with a plastic film in order to prevent any water evaporation. The second slump test measurement was made after 1 h of concrete maturation. In each case, the concrete mix was stirred for one minute before taking the measurements.

#### 2.2.4. Rheology

In order to evaluate the rheology of the cement paste containing 10% SF (characterized by a Blaine-specific surface of 4650 cm^2^/g) as a partial replacement of cement, the water-to-binder ratio (w/b) was assumed to be 0.35. In this case, for an amount of water equal to 35 g, the mix contains 90 g of cement and 10 g of SF, as well as an added superplasticizer (PCE) in an amount of 0–2.5 wt% of the total cement-based binder. The test was performed at a temperature of 20 ± 1 °C. The rheology of the cement paste was evaluated using a VT 550 viscometer, which is a rotational viscometer. Using this viscometer, the following parameters of cement pastes can be measured: shear stresses (τ) and viscosities (µ), which are obtained as a function of shear rates ($\dot{\gamma }$). 

#### 2.2.5. Zeta Potential

The zeta potential represents the measurement of the intensity of electrostatic repulsion or attraction between particles. Therefore, its measurement provides an understanding of the causes of dispersion or flocculation of cement grains in suspensions. The zeta potentials of the cement pastes (containing PCEs at saturation dosages) were determined using a Malvern ZETASIZER 2000 in materials, processes and environment research unit; Algeria. In a standard experiment, 1 cm^3^ of the cementitious suspension is diluted in 30 cm^3^ of distilled water, after which 5 mL of this suspension is injected into the analyzer.

#### 2.2.6. Compressive Strength

In order to determine the compressive strength, six cylindrical concrete specimens (150 × 300 mm^2^) were prepared according to the EN 206-1 protocol. These specimens were kept for 24 h in molds. After demolding, all the specimens were stored in tap water at 20 ± 1 °C for 3, 7, and 28 days.

## 3. Results and Discussion

### 3.1. Molecular Weight Analysis

**Figure 3:**
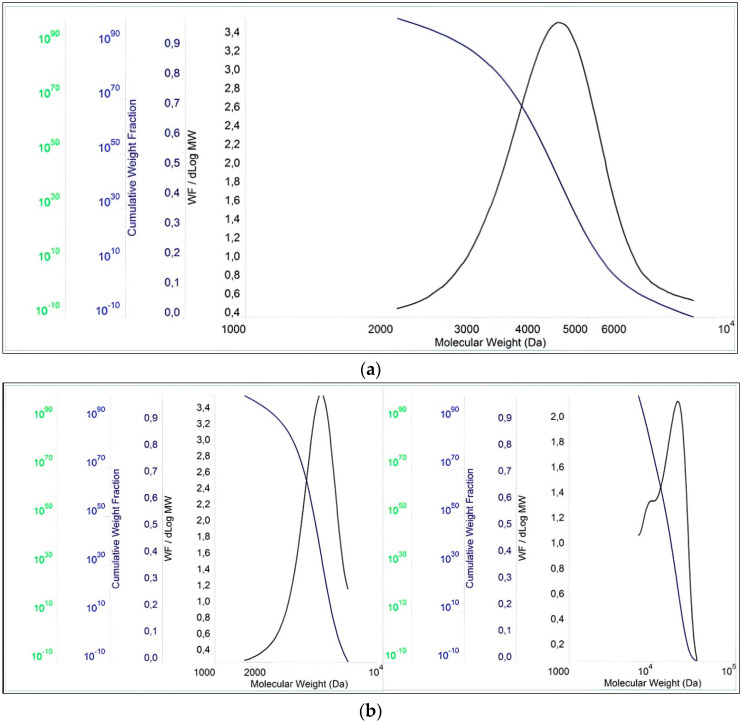

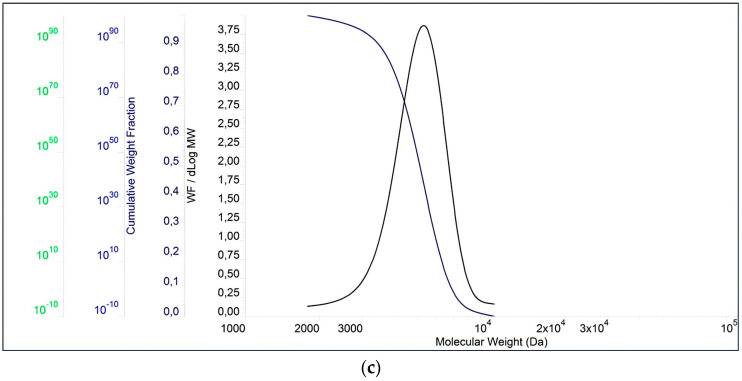
Chromatogram of polymers: (**a**) PCE1, (**b**) PCE2, and (**c**) PCE3.

The molecular properties of the PCEs are shown in Figure 3 and Table 2. The results show that the PDI values for these polymers are slightly above 1.00, which in turn indicates a small dispersion of molecular weights in the polymers. Furthermore, the PDI value of PCE3 is the closest to 1 compared to the other two polymers. This means that PCE3 consists of the most similar macromolecules (regarding their length), which have a similar molar mass. In terms of molecular weight, expressed both as Mn and Mw, PCE2 obtained the highest values, while PCE1 obtained the lowest ones.

### 3.2. Analysis of the Content of the Carboxyl Groups 

The content of the carboxyl groups of the PCEs is shown in Table 2. This content directly influences the carboxyl density of the PCEs. It should be mentioned that both of these values increase simultaneously—the increase in one causes a rise in the other. Therefore, PCE3 has the highest carboxyl density, whereas PCE1 has the lowest one. This is due to the fact that the content of the carboxyl groups in PCE3 and PCE1, expressed for 1 g of the polymer, amounts to 1.95 and 1.80 mmol, respectively. The density of carboxyl groups in the main chain has a significant influence on the adsorption effect of PCE on cement particles. First, the increased density of carboxyl groups can enhance the adsorption capacity of PCEs [12,20]. However, different molecular structures will have different optimal carboxyl densities.

### 3.3. Fluidity

**Figure 4:**
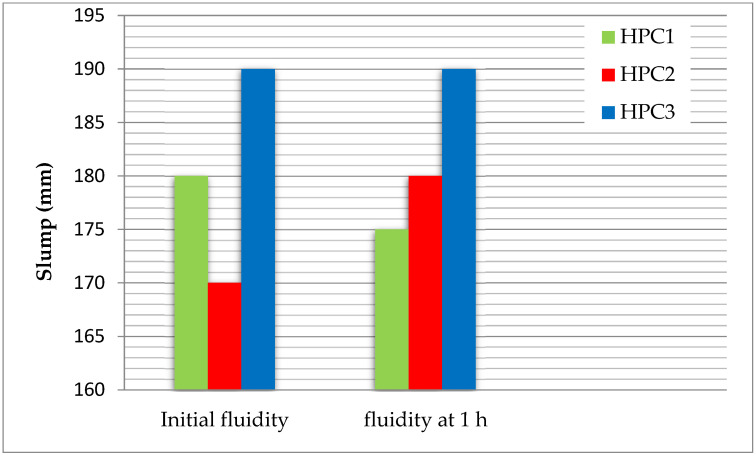
Slump values of the HPC.

The results of the initial fluidity (slump) and the fluidity assessed after 1 h (slump retention) are shown in Figure 4. PCE3, with the highest charge density and a moderate molecular weight, exhibited a higher value of initial fluidity and a higher value of fluidity after 1 h compared to PCE1 and PCE2. The COO– group on the backbone of the PCE was adsorbed on the surface of cement particles by complexation with Ca^2+^ or by electrostatic attraction. An increase in the number of COO– groups increases the adsorption capacity of PCEs [32]. It means that the flowability of the cement paste increased with a rise in the content of the COO– groups. Therefore, very good compatibility was found between PCE3 and the SF, as they both retained the fluidity of the cement pastes after 1 h. 

PCE1, with the lowest carboxylic density and molecular weight, exhibited a poor slump and poor slump retention due to the prior adsorption of the PCE on the silica fume surfaces [22,29]. The SF provided additional surfaces for the adsorption of the PCE, which consequently reduced PCE adsorption on the cement surfaces. Moreover, it reduced the performance of the concrete, and it can be stated that there is an incompatibility between PCE1 and the SF.

PCE2 (with the highest molecular weight) had a bad initial slump and the highest slump retention. The amount of absorbed PCE on the surface of the cement paste increased with a decrease in molecular weight. Therefore, the PCE with a high molecular weight significantly delayed the hydration of the cement. This is due to the fact that the main chain of PCEs adsorbs on different cement particles simultaneously, making them agglomerated and, in turn, hindering the hydration process [33].

### 3.4. Rheology

**Figure 5:**
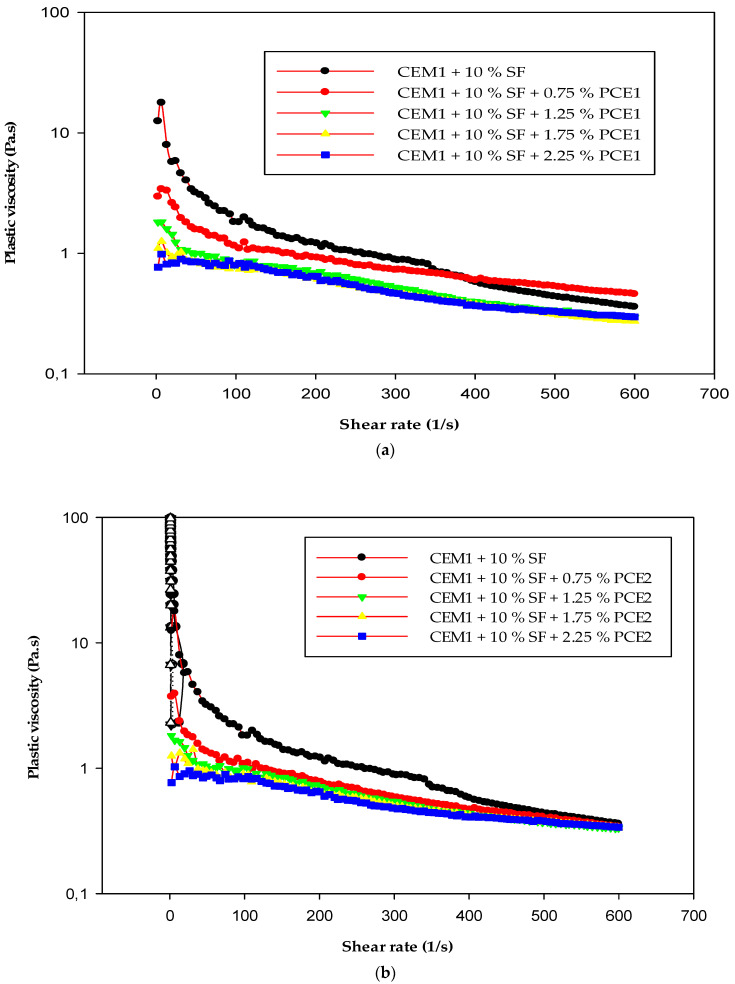

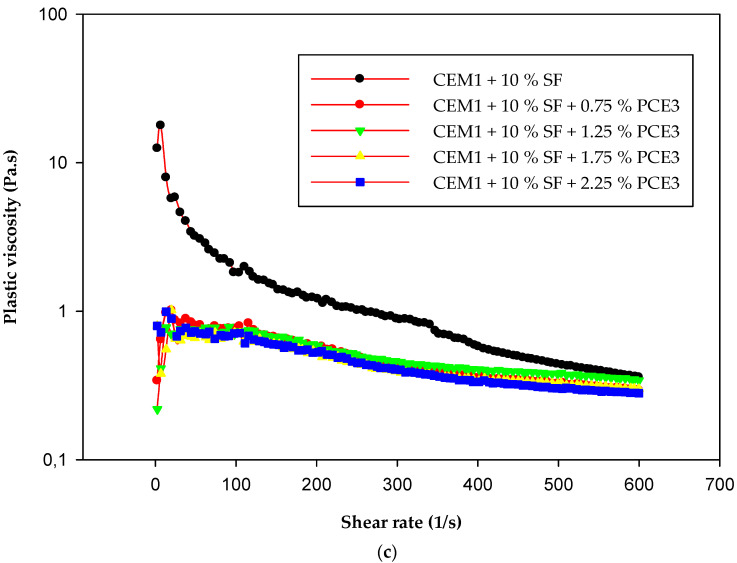
Plastic viscosity as a function of the shear rate of the cement paste: (**a**) PCE1, (**b**) PCE2, (**c**) PCE3; w/b = 0.35.

**Figure 6:**
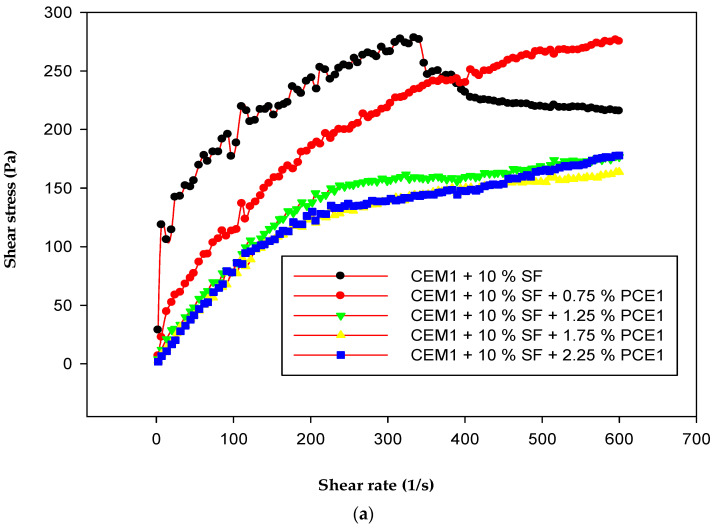

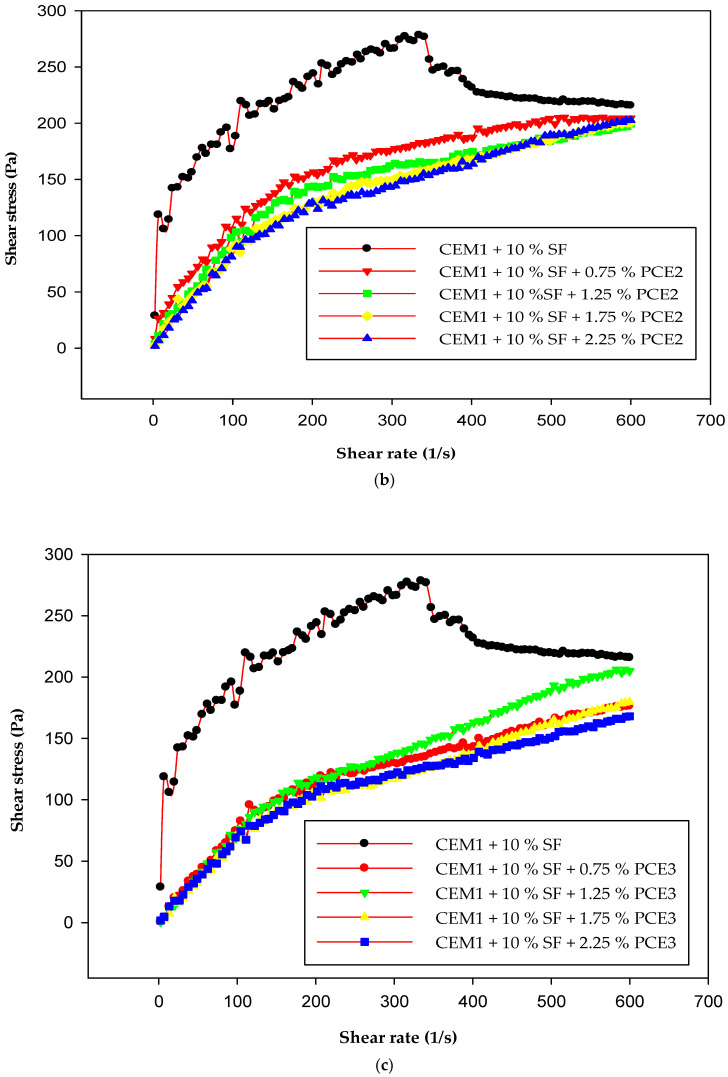
Shear stress as a function of the shear rate of the cement paste: (**a**) PCE1, (**b**) PCE2, (**c**) PCE3; w/b = 0.35.

The saturation point is the value of dosage beyond which the superplasticizer has no effect on the rheological properties of the cement paste. It was determined using the viscometer VT 550. Each of the polymers (PCE1, 2, and 3) had the same saturation point value, i.e., 1.75, at the tested water-to-binder ratio (w/b). Figure 5 and Figure 6 show that shear stress and viscosity decreased with an increase in the dosage of the superplasticizer. The cement pastes became more fluid, with their flow approaching the characteristics of Newtonian flow. The shear stress and viscosity depend on the applied stress. The shear stress increases with a rise in the shear rate, whereas the viscosity decreases with an increase in the shear rate. After adding the PCEs, the yield stress was reduced [34,35]. The cement paste with PCE that has a moderate molecular weight and a high content of carboxylic groups exhibits the lowest shear stress and the lowest plastic viscosity. The carboxylic density and molecular weight of PCEs have a great effect on their dispersing performance. As the carboxylic density increases, the dispersing capability of a PCE improves [10]. PCE3 had the highest carboxylic density and a moderate molecular weight. Due to this, it exhibited the best adsorption behavior on cement grains, which in turn resulted in the lowest viscosity and shear stress of the samples with this polymer. PCE3, which had the highest anionic charge density and a moderate molecular weight, was more compatible with the SF when compared to PCE1 and PCE2. 

### 3.5. Zeta Potential

**Figure 7:**
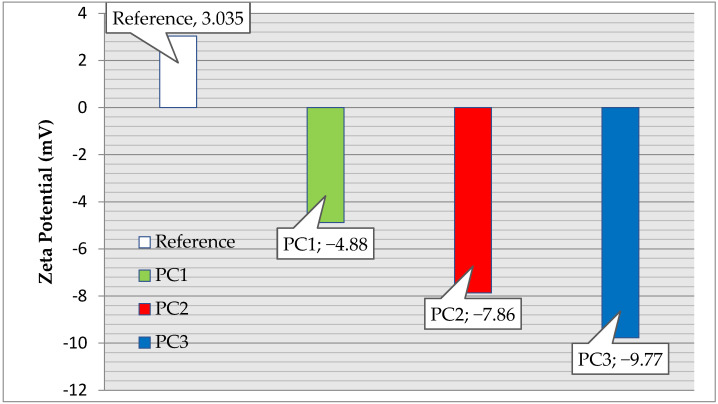
Zeta potential of the cement paste as a function of the PCE type.

The zeta potentials of the cement pastes prepared with the addition of the PCEs (with different carboxylic densities and molecular weights) are shown in Figure 7. The order of the zeta potentials is as follows: reference sample < PCE1 < PCE2 < PCE3. This indicates that there is a good correlation between the zeta potential and carboxylic density. The results show that the zeta potential of the cement paste initially exhibited a positive value (3.035 mV) but then changed to a negative value after adding the PCE. This is because there is adsorption of the anionic groups (COO–) from the PCE on the surface of the hydration products of cementitious materials (via electrostatic adsorption by complexation with Ca^2+^). PCE3 had a high carboxyl content (1.95 mmol/g) and a moderate molecular weight, whereas its polydispersity index (5157 Da, 1.081) presented a high absolute value of zeta potential in the cement paste (9.7735 mV). This is due to its having the best adsorption capacity with regard to cement particles.

### 3.6. Compressive Strength

**Figure 8:**
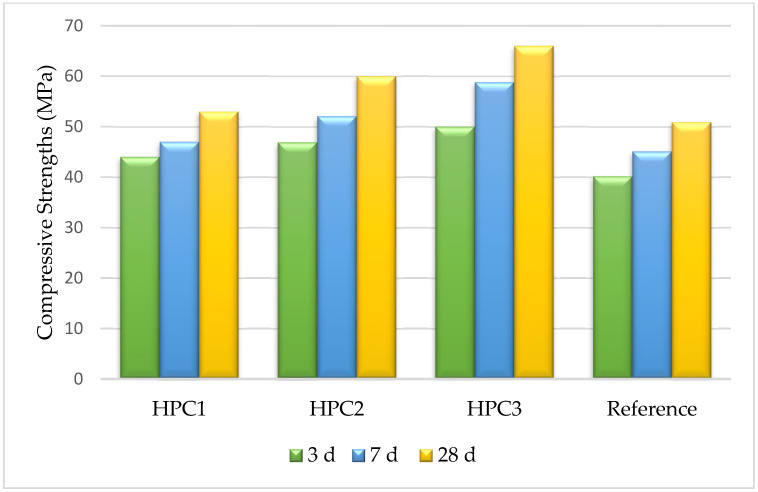
Compressive strengths of the HPC (containing superplasticizers PCE1, 2, and 3) after different times of hydration.

The compressive strengths at 3, 7, and 28 days of HPC without superplasticizers are, respectively, 40.2, 45.1, and 50.9 MPa. They are lower than the compressive strength of the HPC with PCEs (Figure 8). Therefore, the three superplasticizers significantly improve the mechanical behavior of HPC. The compressive strength values of the hardened HPC were found to be the highest for the samples containing PCE3. The compressive strength values obtained for these samples, measured after 3, 7, and 28 curing days, were 50, 58.8, and 66 MPa, respectively. Generally, PCEs with high zeta potential exhibit good compressive strength values, which in turn are correlated with better adsorption of superplasticizer molecules [36].

The aim of this work is to study the compatibility of a polymer with silica fume in HPC. The results show that the chemical structure (molecular weight; carboxylic group) of the polymer influences this compatibility. The best chemical structure is a polymer with a moderate molecular weight and high carboxyl density. 

## 4. Conclusions

- The PCE with a moderate molecular weight and the highest content of carboxylic groups has the best dispersion (high value of zeta potential), the lowest viscosity, and the highest compressive strength of hardened HPC. Therefore, it can be considered to be compatible with SF. Such compatibility was not observed in the case of the PCE with the low carboxyl density, which was due to the fact that this PCE was adsorbed on the SF and reduced the ability of the cement particles to disperse. Therefore, the workability of the HPC was also reduced.
- There is a clear relationship between the zeta potential and rheological properties of cement paste and the anionic charge density and molecular weight of PCEs. A good correlation was also found between zeta potential, carboxylic density, and compressive strength.
- The results from the retention slump tests show that a PCE with a high molecular weight can be applied to precast concrete, whose consistency has to be retained over a longer period of time (until this concrete is delivered to a construction site).
- The molecular weight of polycarboxylate superplasticizer has a great impact on the properties of cementitious systems. Therefore, PCE3 can be seen to be a perfect polymer in which all the macromolecules have the same length and the same molar mass (PDI close to 1). This, in turn, makes it the most efficient admixture, which was proved by the results of the research conducted.

## Figures and Tables

**Table 1:** The analytical conditions of the SEC.

System	Column	Col Temp (C)	Solvent	Flow Rate (mL/min)	Inj Vol (μL)
SEC THF	SDV	35.00	THF	1.0000	100.0

**Table 2:** Properties of the PCEs.

Property	Type of Superplasticizer		
	PCE_1_	PCE_2_	PCE_3_
Dry extract	22 ± 1%	30 ± 1%	30 ± 1%
pH	5.5	4.46	3.35
Density (g/cm^3^)	1.06 ± 0.02	1.07 ± 0.02	1.07 ± 1
Mn (Da)	4036	13,621	4771
Mw (Da)	4386	15,959	5157
PDI	1.087	1.172	1.081
Carboxyl content (mmol/g)	1.80	1.87	1.95

PDI = Polydispersity index. Mn = Number average molecular weight of PCE. Mw = Weight average molecular weight of PCE.

**Table 3:** Chemical composition of the PC and SF.

	PC	SF
Oxide content (%)		
Silicon dioxide (SiO_2_)	22.18	95.5
Aluminum oxide (Al_2_O_3_)	3.72	1.0
Iron oxide (Fe_2_O_3_)	0.17	1.0
Calcium oxide (CaO)	66.55	0.4
Magnesium oxide (MgO)	1.75	0.5
Sulfur trioxide (SO_3_)	2.53	0.1
Potassium oxide (K_2_O)	0.52	-
Sodium oxide (Na_2_O)	0.07	0.6
Sodium oxide Na_2_O Equivalent	0.41	0.4
Mineralogical composition of the cement determined using the Bogue equation		
Tricalcium silicate (C3S)	77.07	-
Dicalcium silicate (C2S)	5.52	-
Tricalcium aluminate (C3A)	9.57	-
Tetracalcium aluminoferrite (C4AF)	0.52	-
Physical properties		
Specific gravity (g/cm^3^)	3.07	2.3
Bulk density (g/cm^3^)	0.97	0.25
Blaine fineness (cm^2^/g)	3711.89	230.000

**Table 4:** The properties of the aggregates.

Aggregates		Fineness Modulus	Sand Equivalent (%)	Apparent Density (kg/m^3^)
Fine Aggregate	Sand 0/1	1.14	83.5	1590
	Sand 0/4	2.64	74.5	1540
Coarse Aggregate	Gravel 8/15	----	----	1480
	Gravel 15/25	----	----	1470

**Table 5:** The formulations of the concrete mixes.

PC (kg/m^3^)	SF (kg/m^3^)	Coarse Aggregate (kg/m^3^)	Fine Aggregate (kg/m^3^)	Water (kg/m^3^)	PCE (kg/m^3^)	w/b
382.5	42.5	1031.5	810.5	157.5	7.45 PCE1	0.35
382.5	42.5	1031.5	810.5	157.5	7.45 PCE2	0.35
382.5	42.5	1031.5	810.5	157.5	7.45 PCE3	0.35

## Data Availability

Not applicable.
